# Fatal air embolism caused by air entry into the cardiopulmonary bypass circuit during minimally invasive atrial septal defect repair: a case report

**DOI:** 10.3389/fcvm.2025.1660416

**Published:** 2025-08-29

**Authors:** Jin Zeng, Yuanyuan Yang, Jing Xiao

**Affiliations:** ^1^ Department of Anesthesiology, First Affiliated Hospital of Guangxi University of Science and Technology, Liuzhou, China; ^2^ Hospital Office, First Affiliated Hospital of Guangxi University of Science and Technology, Liuzhou, China

**Keywords:** three-port thoracoscopic minimally invasive surgery, congenital heart disease, atrial septal defect repair, cardiopulmonary bypass, severe complications, cerebral air embolism

## Abstract

An 18-year-old female patient was preoperatively diagnosed with atrial septal defect (ASD) and tricuspid regurgitation. The patient's had a complex secundum-type superior sinus venosus ASD, rather than a primum ASD, which was anatomically unsuitable for transcatheter closure. The patient underwent minimally invasive ASD repair under general anesthesia using three-port totally thoracoscopy with cardiopulmonary bypass (CPB). During the initiation of CPB, poor venous drainage through the right internal jugular vein cannula allowed air to enter the CPB circuit, leading to intraoperative cardiac arrest. Immediate cardiopulmonary resuscitation, intravenous administration of epinephrine, and other emergency interventions were carried out. The procedure was urgently converted to conventional median sternotomy, in order to reestablish CPB and complete the ASD repair. After the operation, the patient developed extensive cerebral air embolism, cerebral edema, brain herniation, and multiorgan failure, including respiratory and circulatory collapse, ultimately resulting in death. This case report analyzes the possible causes of air entry into the CPB system, cerebral air embolism, and the patient's death, aiming to draw lessons to prevent similar serious adverse events, and improve the safety of cardiovascular surgeries.

## Introduction

Conventional open-heart cardiovascular surgeries and minimally invasive three-port totally thoracoscopic procedures are typically performed under cardiopulmonary bypass (CPB), with the heart arrested or beating. CPB remains as the primary approach in cardiovascular surgeries. Compared to traditional median sternotomy, three-port totally thoracoscopic minimally invasive surgery demonstrates significant advantages, including shorter operative duration, reduced CPB time, less intraoperative blood loss, faster postoperative recovery, decreased intensive care unit (ICU) stay, and shorter hospitalization time (1–4). However, the CPB system used in cardiovascular surgery involves complex operations and multiple tubing connections, requiring a high level of technical expertise for proper management. In critical situations, improper or delayed management of CPB-related complications or equipment malfunctions during cardiovascular surgery can lead to severe outcomes, including permanent disability or death.

## Case presentation

An 18-year-old female patient was admitted to our hospital following the detection of congenital heart disease during a routine physical examination. The patient was asymptomatic, with no complaints of palpitations, shortness of breath, headache, or dizziness. The initial diagnosis included the following: (1) congenital heart disease: atrial septal defect (ASD); (2) tricuspid regurgitation, with right atrial and right ventricular enlargement, and cardiac function grade II. The transthoracic echocardiography revealed a 20 mm ASD. The patient had a complex secundum-type superior sinus venosus ASD, rather than a primum ASD, which was anatomically unsuitable for transcatheter closure. The patient underwent all preparations for cardiac surgery. The minimally invasive ASD repair was planned using three-port totally thoracoscopy under bronchial intubation general anesthesia, and performed on a beating heart with CPB.

At six days after admission, following the induction of general anesthesia via bronchial intubation using standard methods, venous drainage access was established through cannulation of the right internal jugular vein and right femoral vein. Arterial access was achieved via right femoral artery cannulation. Thus, an extracorporeal circulation circuit with the right internal jugular vein, right femoral vein, and right femoral artery was established. After cannulation of the right internal jugular vein, right femoral artery, and right femoral vein was completed. The patient's preoperative activated clotting time (ACT) was 178 s. After intravenous administration of standard-dose heparin for heparinization, the ACT increased to 501 s. Before initiating CPB, the perfusionist, anesthesiologist, and surgeon completed a 22-item checklist, which included verification of: patient information and surgical procedure, power supply status, patency of oxygen pipelines, oxygen concentration, gas balance, CPB machine pump head settings and operation, manual crank functionality, oxygenator integrity, pipeline connections and de-airing, water bath working status and controller settings, level alarm connection and functional, three-channel pressure monitoring and zero calibration, preparation of cannulas and connectors, direction and negative pressure of left and right heart suction, and ACT greater than or equal to 480 s, etc. CPB was initiated. However, inadequate drainage was observed from the right internal jugular vein cannula. Color Doppler ultrasound was then used to adjust and verify the localization and direction of catheterization for the right internal jugular vein, right femoral artery and right femoral vein, confirming correct placement. Subsequently, the CPB machine was restarted. The patient's blood pressure gradually decreased, and the heart rate slowed. Two intravenous injections of 0.50 mg epinephrine were administered, resulting in a temporary elevation of blood pressure and an increase in heart rate. Subsequently, the blood pressure rapidly dropped to 30/20 mmHg, and the heart rate declined to 30 bpm. Approximately 40 s later, cardiac arrest occurred.

The patient was immediately repositioned from the left lateral decubitus to the supine position, and external chest compressions were initiated. Three intravenous injections of 1 mg epinephrine were administered, but cardiac activity was not restored. While continuing chest compressions, an emergency median sternotomy was performed, followed by cannulation of the ascending aorta, superior vena cava, and inferior vena cava. CPB was re-established, and high-potassium cardioplegia solution was infused via the aortic root into the coronary sinus for myocardial protection. Then, the surgical team proceeded with the ASD repair under conventional CPB. At the time CPB was restarted, the patient's pupils were bilaterally dilated to 4.00 mm with sluggish light reflexes. These signs may be related to severe hypotension during the emergency re-establishment of CPB after cardiac arrest, and to the possible cerebral air embolism that may have occurred during the initial initiation of CPB. Therefore, a reverse Trendelenburg position (head-low, feet-up) was immediately adopted during resuscitation. Meanwhile, 100 mg of hydrocortisone was intravenously administered, and 500 mg of methylprednisolone was added to the CPB circuit.

Following the successful establishment of CPB, the surgery proceeded uneventfully, and the CPB was successfully weaned. After weaning, the patient was maintained on continuous intravenous infusion of dopamine at 6 μg/kg/min and nitroglycerin at 0.1 μg/kg/min. Postoperatively, the patient was transferred to the ICU for close monitoring.

Upon ICU admission, the patient's vital signs, including blood pressure, heart rate, pulse, and oxygen saturation, were within the normal range. However, bilateral pupil dilation to 5.00 mm was observed, with no pupillary light reflex. On postoperative day one, the patient remained in a comatose state. She was transferred to the ICU and underwent an emergency computed tomography scan, which revealed hypodense areas in multiple brain regions. Transcranial Doppler ultrasound monitoring showed reduced blood flow velocities in the anterior cerebral artery, middle cerebral artery, and posterior cerebral artery, with the most significant reduction observed in the middle cerebral artery, which also exhibited an abnormally elevated pulsatility index. Subsequent MRI confirmed extensive cerebral infarction secondary to cerebrovascular air embolism, accompanied by severe cerebral edema. The neuroprotective management in the ICU included dehydration therapy, diuretics, and mild hypothermia. Despite these interventions, the patient developed progressive respiratory and circulatory failure, culminating in multiorgan failure. On postoperative day four, the patient experienced cardiac arrest, resulting in death.

## Discussion

Iatrogenic air embolism is a complication caused by the entry of air into the vascular system during medical procedures. Although relatively rare, delayed diagnosis and treatment can result in cardiac arrest and severe complications, including permanent disability or death (5, 6). Iatrogenic air embolism can be classified as arterial and venous types. The most fatal outcome of venous air embolism is pulmonary embolism, while arterial air embolism can be life-threatening when involving coronary or cerebral arteries, with a mortality rate of 26.30% (7).

Iatrogenic air embolism may occur during both conventional open-heart surgery with CPB and minimally invasive three-port totally thoracoscopy. With the advancements in medical technology, its incidence has decreased to 0.02%–0.07%. However, the complexity and high technical demands of the CPB system require well-trained and experienced perfusionists to ensure safe operation.

During CPB, any unexpected event, such as cannula dislodgement, occlusion of the perfusion system causing pipe rupture, CPB pump malfunction, impaired venous return requiring excessive negative pressure, or abnormal low fluid levels in the oxygenator, can introduce a significant volume of air into the CPB circuit, potentially resulting in multiorgan air embolism. If not properly managed, such events can cause severe complications, including tissue ischemia and hypoxia. In some cases, patients may suffer from cardiac and respiratory arrest, hypoxic-ischemic encephalopathy, persistent vegetative state, or even death.

The three-port totally thoracoscopy-assisted repair of congenital heart defects, such as ASD, and ventricular septal defects using a beating-heart approach is an innovative minimally invasive surgical technique (8). Compared to traditional median sternotomy, this approach is associated to less surgical trauma, since the heart can be operated without cardioplegic arrest, leading to faster postoperative recovery. Although more invasive than percutaneous device closure, thoracoscopic surgery prevents the need for the long-term anticoagulation required for device implantation. Furthermore, ASDs larger than 34 mm, primum ASDs, sinus venosus ASDs, or severe pulmonary hypertension with right-to-left shunt are contraindications for percutaneous device closure, and must be treated with surgical repair (minimally invasive or conventional surgery). Therefore, thoracoscopy-assisted repair offers distinct advantages in managing large or complex ASDs.

In the present case, during the initiation of CPB, venous drainage through the right internal jugular vein cannula was inadequate. Despite three attempts to reposition the cannula, drainage could not be restored. However, the venous drainage through the femoral vein remained unobstructed. At that time, a large volume of air bubbles was observed in the femoral artery cannula. The patient experienced a rapid decline in blood pressure and a reduction in heart rate. Immediate measures, including placing the patient in the Trendelenburg position and administering two intravenous injections of 0.50 mg epinephrine, were undertaken, which temporarily stabilized the blood pressure. However, both the blood pressure and heart rate continued to drop, culminating in cardiac arrest. The patient was immediately repositioned to the supine position. External chest compressions were initiated, and intravenous epinephrine was administered. Then, emergency median sternotomy was performed. Subsequently, cannulation of the ascending aorta, superior vena cava, and inferior vena cava was rapidly established to initiate conventional CPB. Cardioplegia was administered via the aortic root to arrest the heart, and protect the myocardium, enabling the successful completion of the ASD repair.

Several mechanisms may account for the entry of air into the CPB circuit in the present patient: (1) A significant amount of air may not have been completely evacuated from the venous drainage lines of the internal jugular and femoral veins before initiating CPB. (2) During CPB initiation, venous drainage via the right internal jugular vein was obstructed. Under the strong negative pressure generated by the drainage pump, air may have entered through the suture sites of the internal jugular or femoral venous cannulas, into the venous system. The large volume of air introduced into the venous could have been retrogradely carried under high negative pressure into the superior or inferior vena cava, and subsequently into the right atrium. Due to the presence of ASD, the air could have shunted from the right atrium to the left atrium, entered the left ventricle, and been ejected into the systemic arterial circulation via the aorta. Once in the arterial system, the air could have traveled through various arteries, resulting in air embolism in multiple organs. For instance, air entering the aortic arch may have reached the brain via the carotid and vertebral arteries, leading to extensive cerebral air embolism. Furthermore, air may have entered the coronary, pulmonary, or other systemic arteries, causing myocardial air embolism, pulmonary embolism, and embolic events in other organs. In the present patient, the persistent obstruction of venous drainage through the internal jugular vein likely allowed a significant volume of air to enter both the left and right coronary arteries, resulting in myocardial ischemia, impaired cardiac contractility, and suppression of the cardiac conduction system, ultimately causing a rapid decline in both blood pressure and heart rate, followed by cardiac arrest. The present patient experienced an intraoperative air embolism during CPB, which led to extensive cerebral infarction, hypoxic-ischemic encephalopathy, severe cerebral edema, and intracranial hypertension. Despite the implementation of mild hypothermia and dehydration therapy, the patient developed cerebral herniation on postoperative day four, resulting in cardiac arrest and multiorgan failure, and ultimately death. The patient was transferred to the ICU with an endotracheal tube in place. Due to the absence of spontaneous respiration and hemodynamic instability requiring vasoactive agents to maintain blood pressure, the patient remained critically ill. Unfortunately, none of the hospitals in our prefecture-level region were equipped with hyperbaric oxygen chambers capable of accommodating intubated patients. As a result, the patient was unable to receive timely hyperbaric oxygen therapy after surgery.

In the present case, a large volume of air may have inadvertently entered the CPB circuit, and been carried into the right atrium. Due to the presence of ASD, the air may have passed through the left atrium and left ventricle, and subsequently entered the aorta, causing severe multiorgan air embolism. The present case provides valuable insights in clinical management: (1) The successful implementation of cardiovascular minimally invasive procedures requires a well-coordinated and experienced team, including cardiothoracic surgeons, anesthesiologists, and nurses. Immature techniques can increase the risk of serious complications. (2) Before initiating CPB, the perfusionist, anesthesiologist, and surgeon completed a 22-item checklist to ensure safety. (3) If poor venous drainage occurs, negative suction should be immediately halted. The cannulas and tubing must be inspected and adjusted to prevent adherence to the vessel wall. Increasing negative pressure in the presence of loose venous cannulation, especially with ASD, may draw air into the venous system and CPB circuit, resulting in air emboli passing into the systemic circulation, causing embolism in multiple organs, such as the heart, lungs, brain, liver and kidneys, and leading to life-threatening complications or even death. (4) When repeated repositioning of the right internal jugular vein catheter fails to restore adequate drainage, the procedure should be converted to a conventional median sternotomy. Early conversion may improve outcomes. This is a painful lesson drawn from this unfortunate event. (5) If a significant amount of air is detected in the CPB circuit or arterial line, immediate interventions must be initiated for cerebral, cardiac, and pulmonary air embolism. For suspected cerebral embolism, placing the patient in the Trendelenburg can reduce air entry into the brain (9). High-pressure arterial perfusion through CPB or retrograde perfusion via the internal jugular vein may help displace the air from cerebral arteries or the microcirculation. Meanwhile, mild hypothermia, hyperbaric oxygen therapy, and diuretics should be used for brain protection. (6) Transesophageal echocardiography is the gold standard for detecting cardiac and pulmonary artery emboli (10). Its location, size, and quantity can be monitored through the ultrasonic images of great cardiac vessels. (7) When clinical manifestations suggest coronary air embolism, coronary computed tomography angiography (CTA) can be used to determine the extent and location of the emboli in the coronary arteries (11). (8) Cerebral blood flow and oxygenation can be assessed using cerebral oximetry or transcranial Doppler ultrasound, which is valuable for diagnosing cerebral air embolism, and evaluating the prognosis (12).

In conclusion, the present case report presents a patient with ASD, who suffered an iatrogenic cerebral air embolism and subsequent death following minimally invasive thoracoscopic surgery with CPB. In the management of cardiovascular diseases, such as congenital heart defects (e.g., ASD and ventricular septal defects), valvular heart diseases (e.g., aortic or mitral stenosis), and aneurysms (e.g., thoracic or abdominal aortic aneurysm), the choice of surgical approaches, whether interventional, thoracoscopic, or open, must strictly follow the indications and contraindications, with patient safety as the top priority. The use of novel surgical techniques requires not only rigorous professional training, but also well-established crisis response protocols and routine emergency drills. In other words, the whole team should strengthen specialized education and intensive training in newly adopted surgical techniques, improve interdisciplinary collaboration, and enhance the implementation of emergency response protocols.

## Data Availability

The original contributions presented in the study are included in the article/Supplementary Material, further inquiries can be directed to the corresponding author.
